# Secondary analysis reveals gut microbiota differences in patients with Parkinson’s disease and/or cognitive impairment

**DOI:** 10.20517/mrr.2024.35

**Published:** 2024-08-28

**Authors:** Xin Shen, Bing Leng, Shukun Zhang, Lai-Yu Kwok, Feiyan Zhao, Jia Zhao, Zhihong Sun, Jinbiao Zhang

**Affiliations:** ^1^Inner Mongolia Key Laboratory of Dairy Biotechnology and Engineering; Key Laboratory of Dairy Products Processing, Ministry of Agriculture and Rural Affairs; Key Laboratory of Dairy Biotechnology and Engineering, Ministry of Education, Inner Mongolia Agricultural University, Hohhot 010018, Inner Mongolia, China.; ^2^Department of neurology, Weihai Municipal Hospital, Cheeloo College of Medicine, Shandong University, Weihai 264200, Shandong, China.; ^3^Shandong Probincial Key Medical and Health Laboratory of Geriatric Gastrointestinal Tumor Pathology, Department of Pathology, Weihai Municipal Hospital, Cheeloo College of Medicine, Shandong University, Weihai 264200, Shandong, China.; ^#^Authors contributed equally.

**Keywords:** Parkinson’s disease, cognitive impairment, gut microbiota, hippocampus

## Abstract

**Background:** Parkinson’s disease (PD) is a neurodegenerative disorder, and the main clinical characteristics are bradykinesia and muscle stiffness. Cognitive impairment (CI) is a prevalent non-motor manifestation observed in individuals with PD. According to disease severity, it can be divided into PD with mild cognitive impairment (MCI) and PD dementia. CI in PD patients may precede motor symptoms, and the gut microbiota plays an important role in PD pathogenesis. Therefore, gut microbiota may be one of the diagnostic targets for PD-CI.

**Methods:** This study compared the gut microbiota of 43 PD-CI patients [Montreal Cognitive Assessment (MoCA) score < 26] and 38 PD patients without CI (MoCA ≥ 26). Patients’ neuropsychological conditions, depression scale, and brain structure scanned by magnetic resonance imaging (MRI) were also recorded. The fecal metagenomic datasets of patients with PD, PD-CI, and CI only were retrieved from public databases for reanalysis to explore the relationship between PD, CI, and gut microbiota.

**Results:** We found that the cortical thickness and the volume of the hippocampus, gray matter, and thalamus were significantly reduced among patients with PD-CI compared to PD without CI (*P* < 0.05). Moreover, the gut microbiome in patients with PD-CI had fewer short-chain fatty acid (SCFA) producing bacteria and more pathogenic bacteria. There were also alterations in patterns of metabolic pathway-encoding genes. Additionally, PD affected gut microbiota more than CI.

**Conclusion:** CI may aggravate the severity of PD, but it did not drastically alter subjects’ gut microbiota. This study reveals the relationship between gut microbiota, PD, and CI.

## INTRODUCTION

Parkinson’s disease (PD) is a prevalent neurodegenerative condition that commonly affects individuals of middle to old age groups. In addition to motor symptoms such as bradykinesia, and postural balance impairments, PD patients may experience non-motor symptoms including mild cognitive impairment (MCI), dementia, sleep disorders, anxiety, depression, and autonomic dysfunction^[1]^. Studies found that non-motor symptoms indeed occur earlier than motor symptoms in patients with PD^[2]^. Therefore, PD is also considered a multi-system disease.

It is worth mentioning that cognitive impairment (CI) plays a significant role in the manifestation of non-motor symptoms in PD, which are characterized by cognitive slowing, impaired abstract thinking, and reasoning difficulties. All of these conditions directly affect a patient’s quality of life. Now, the prevalence of PD is on the rise, and its prevalence is expected to increase to 12-17 million people by 2040^[3]^. It is reported that about 1% of middle-aged and elderly people suffer from PD^[4]^. PD with dementia is one of the biggest risk factors for death in PD patients, and dementia occurs in up to three-quarters of PD patients^[5]^. Furthermore, there is evidence that the prevalence of dementia among individuals with PD is between 15%-20% within the first 5 years and increases to 46% after 10 years^[6,7]^. At present, the etiology of PD is considered to be heterogeneous and also idiopathic^[8]^. Studies suggest that the risk factors for PD and CI are age, environment, and genetics. The pathogenesis for PD involves oxidative stress, glutamate excitotoxicity, mitochondrial dysfunction, and neurotrophic factor deficiency^[9]^.

Another major element in the development of disorders like PD and cognitive dysfunction disorder is gut microbiota, which affects mood, memory, cognition, and behavior of the host through the bi-directional “gut-brain axis”. Research has shown that PD patients have abundant *Lactobacillus*, *Akkermansia*, and *Bifidobacterium* as their gut microbiota, but are deprived of important short-chain fatty acid (SCFA)-producing bacteria, such as those from *Faecalibacterium* genus and Lachnospiraceae family^[10]^. A cohort study found that the gut microbiota composition of PD patients was different compared to control groups, and also the carbohydrate metabolism and lipid metabolism pathways were damaged^[11]^. Moreover, CI and gut microbiota are observed to be linked with each other. Negative correlations were found between CI and the prevalence of *Odoribacter*, *Butyrivibrio*, and *Bacteroides*, and a positive correlation between *Odoribacter* and hippocampal volume in the brain^[12]^. A previous study has shown that compared to healthy people, the gut microbiota of PD patients with mild CI differs, and is characterized by more *Porphyromonadaceae* but fewer *Blautia* and *Ruminococcus*^[13]^. Similarly, another study reported that *Porphyromonadaceae* is related to poor cognitive ability^[14]^.

Alpha-synuclein (α-syn) is a protein that plays a significant role in the pathophysiology of PD. This protein is not only present in the brain but also in neurons in the intestinal plexus. Therefore, it was postulated that PD could potentially originate in the intestine due to the aggregation, accumulation, and transmission of α-syn from the enteric nervous system to the substantia nigra and other nuclei in the central nervous system via the vagus nerve^[15]^. Pathological features of PD include a large accumulation of misfolded α-syn protein in the brain and the loss of dopaminergic neurons in substantia nigra. The gut microbiota can accelerate the deposition of misfolded α-syn protein, resulting in neurological dysfunction through the gut-brain axis^[16]^. It has been shown that the transplantation of the feces of young and old mice into the intestine of aging mice could alleviate cognitive behavior disorders^[17]^. Transplantation of feces from normal mice to 1-methyl-4-phenyl-1,2,3,6-tetrahydropyridine (MPTP)-induced PD mice could alleviate neuroinflammatory responses and improve motor deficits, while transplantation of feces from PD mice to healthy mice could cause neuroinflammation in normal mice^[18]^. Probiotics administration could also regulate the host metabolism of lipids, SCFAs, and neurotransmitters. It also improves serum dopamine levels and improves PD symptoms, possibly through the modulation of gut microbiota^[19]^. These findings support that gut microbiota is closely linked to degenerative neurological conditions. Therefore, the investigation of the relationship between gut microbiota and non-motor symptoms in PD may offer insights into the development of treatment methods and prevention strategies for the disease. Additionally, targeted gut microbiota modulation may represent a viable strategy to ameliorate or prevent further decline in motor and cognitive functions in PD patients. Although the pathophysiology and development of PD can potentially be influenced by gut microbiota, there is still a knowledge gap on the mechanisms governing this process, especially its involvement in cognitive dysfunction.

By comparing data from PD patients with and without CI, this study aims to evaluate the role of gut microbiota in CI in patients with PD. The included data were obtained from several neuropsychological questionnaires that are used to assess PD and CI, magnetic resonance imaging (MRI), and fecal metagenome sequences. The results of this study provide evidence that a changed gut microbiome is linked to CI in PD patients.

## METHODS

### Participant recruitment

This study was a follow-up work of a previously completed clinical trial^[19]^. In the preliminary trial, we used probiotics Probio-M8 as an intervention for PD in 82 patients, which was given for 3 months. We found that probiotics improved the disease symptoms by regulating the metabolism of lipids, SCFAs, and neurotransmitters. It also increased serum dopamine levels. However, the effect of CI was not considered in that study. Therefore, in the present study, individuals diagnosed with PD were categorized based on CI to investigate the influence of CI on PD. The study used the Movement Disorder Society Clinical Diagnostic Criteria for PD. Patients who were conscious and were able to complete examinations, questionnaires, and provide medical history by themselves or with assistance from their family members were included. All patients were required to sign an informed consent form to be eligible for the research^[19]^. Criteria for participant exclusion were: (1) severe CI that affected words expression either verbally or in writing; (2) profound dysarthria or aphasia; (3) mental unfitness; (4) affected with serious systematic diseases, such as aberrant liver or kidney functions; (5) unavailability of complete information; and (6) unwilling to participate.

**Figure 1:**
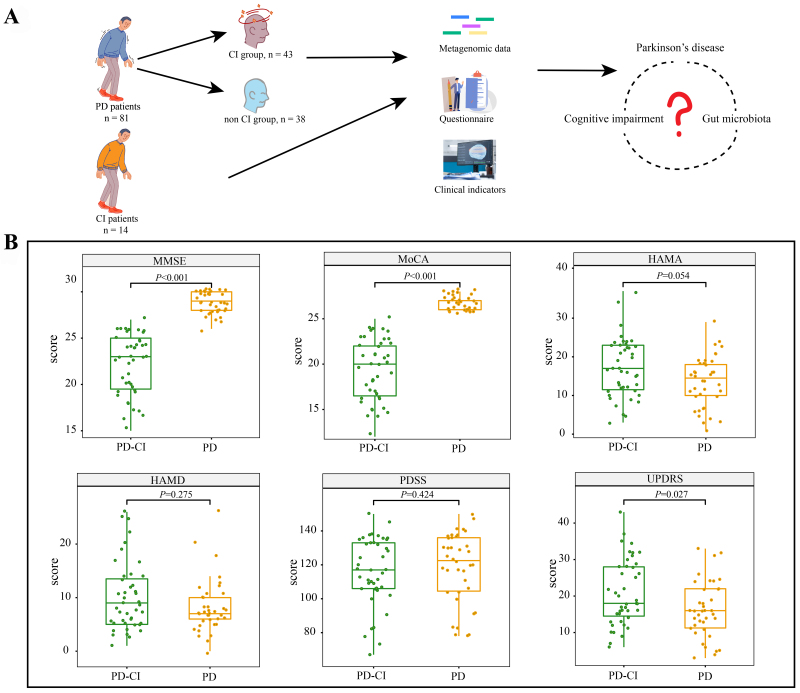
Study design and clinical indicators. (A) Schematic diagram showing the study design; (B) Differences in clinical scores of PD obtained from various neuropsychological questionnaires. Significant differences were seen in Wilcoxon tests; the corresponding *P* values are reported. The boxplots show median values with upper and lower quartiles. MMSE: Mini-Mental State Examination; MoCA: Montreal Cognitive Assessment; HAM-A: Hamilton Anxiety Rating Scale; HAMD: Hamilton Depression Scale; PDSS: Parkinson’s Disease Sleep Scale; UPDRS: Unified PD Rating Scale; PD: PD patients with normal cognition (MoCA ≥ 26); PD-CI: PD patients with cognitive impairment (MoCA < 26).

Finally, 81 participants diagnosed with PD were included. They were divided into PD group (without CI, *n* = 38) and PD-CI group (with CI, *n* = 43). Data obtained from patients’ clinical questionnaires and MRI were combined with previous metagenomic data for analyses [Figure 1A].

### MRI scanning

A 3.0-T magnetic resonance scanner (Siemens Healthcare GmbH, Erlangen, Germany) was used for scanning and data processing of MRI. Specific parameters were assessed based on a previous study^[20]^. The volumes of brain compartments, including the hippocampus, thalamus, cortical thickness, and total gray matter volumes, were measured using the FreeSurfer image analysis suite (version 5.3.0), a freely accessible tool from http://surfer.nmr.mgh.harvard.edu/. The existence of hyperintensity in the white matter region on FLAIR images was designated as White Matter Hyperintensity (WMH). A neurologist used the semiautomated 3D-slicer freeware program (http://www.slicer.org) to quantitatively examine the volumes of periventricular hyperintensity (PVH), deep white matter hyperintensity (DWMH), and deep white matter hyperintensity. A neuroradiologist who was blinded to the clinical details examined each scan for the study.

### Clinical parameters and neuropsychological assessment

The main purpose of this research was to investigate how the gut microbiota differed in PD patients with and without CI. Therefore, the clinical symptoms of recruited patients were assessed using several clinical questionnaires, including: The Unified PD Rating Scale-III (UPDRS-III) to assess overall PD condition, the Mini-Mental State Examination (MMSE), MoCA, Hamilton Anxiety Scale (HAM-A), and Hamilton Depression Scale-17 (HAMD-17) to evaluate mental status, and the Parkinson’s Disease Sleep Scale (PDSS) to quantify sleep quality. Experienced clinicians completed questionnaires to ensure reproducibility and accuracy.

### Data source

Fecal metagenomic sequence raw data of PD patients with and without CI were taken from our previous work, which were retrieved from the National Center for Biotechnology Information (NCBI) Sequence Read Archive (SRA) database (accession number PRJNA769968). The following criteria were used to include CI patients without PD: (1) MoCA < 26; (2) no PD or no other major mental disorders. Finally, the metagenomic data of 14 CI patients without PD were retrieved from the NCBI Genome BioProject PRJNA679346.

### Metagenomic dataset quality control

The downloaded raw data underwent quality checks using the KneadData quality control pipeline (http://huttenhower.sph.harvard.edu/kneaddata; v0.7.5). Low-quality data were filtered using Trimomatic, a flexible trimmer for Illumina-generated sequence data. Human-contaminated sequences were removed using Bowtie2 (v2.3.5.1) before subsequent analysis.

### Metagenomic assembly, taxonomic annotation and abundance of species-level genomic bins

After assembling the data into contigs using MEGAHIT (ver. 1.0), contigs larger than 2,000 bp were selected using VAMB (ver. 1.0) with default settings. These were then assembled to obtain metagenome-assembled genomes (MAGs)^[21]^. Then, species-level genomic bins (SGBs) were extracted from the pool of single representative genomes. Species annotations and relative abundance calculations were performed based on the methodology outlined by Liu *et al.*^[22]^.

### Prediction of relevant gut metabolic modules and bioactive metabolites

The SGBs encoding corresponding gut metabolic module (GMM) in each SGB were predicted using the published literature and the MetaCyc metabolic database^[23,24]^. The predicted open reading frames (ORFs) were then compared with the Kyoto Encyclopedia of Genes and Genomes (KEGG) Orthologues (KOs) database to annotate important metabolic modules. Using SEQTK (https://github.com/lh3/seqtk), one million reads per sample were retrieved, and DIAMON was used to compare results. Then, the gene abundance distribution was calculated for each sample based on the best hit rate for each gene. The gene abundance profiles were converted to predicted bioactive metabolite profiles using the MelonnPan-predict pipeline^[25]^.

### Statistical analyses

Statistical analyses were conducted using R software. Principal coordinate analysis (PCoA) was used to assess microbial community structure differences. The α-diversity (Shannon and Simpson indices) were used to evaluate the diversity and richness of gut microbiota, and the adonis *P*-value was produced using 999 permutations. The Wilcoxon test was employed to evaluate the discrepancies in questionnaire outcomes between groups, MRI findings, gut microbiota composition, and predicted bioactive substances.

## RESULTS

### Demographic data

Eighty-one patients diagnosed with PD were enrolled in the study. Patients were classified into two groups based on their Montreal Cognitive Assessment (MoCA) scores: the PD group (without CI, *n* = 38, MoCA score ≥ 26) and the PD-CI group (with CI, *n* = 43, MoCA score < 26) [Table 1]. Patients in the PD-CI group were slightly older than those in the PD group (69.0 ± 6.7 years *vs.* 65.5 ± 7.0 years; Wilcoxon test, *P* = 0.076). Disease severity was measured by the Hoehn & Yahr (H&Y) staging scale, which shows the progression of disease over time^[26,27]^. However, no significant differences were observed in the diagnosed duration of PD (4.5 ± 2.2 years *vs.* 4.7 ± 2.2 years; *P* = 0.62) or the level of H&Y classification (2.1 ± 0.7 *vs.* 2.0 ± 0.6; *P* = 0.31). Notable differences were observed in the number of years of education between the PD-CI and PD groups (8.6 ± 2.3 years and 12.3 ± 2.2 years, respectively; Wilcoxon test, *P* < 0.001).

**Table 1:** Patient demographics and clinical questionnaires

**Parameters**	**Study groups**		***P***	**Significant difference, Wilcoxon test**
	**PD-CI**	**PD**		
No. of patients	43	38		
Age (years)	69.07 ± 6.73	65.79 ± 7.09	0.0763	Non-significant
Years of formal education	8.65 ± 2.39	12.32 ± 2.26	< 0.0001	****
Duration of confirmed diagnosis of PD	4.55 ± 2.29	4.76 ± 2.21	0.6159	Non-significant
H&Y staging scale	2.15 ± 0.70	2.01 ± 0.63	0.3066	Non-significant
MMSE	22.19 ± 3.30	28.84 ± 1.15	< 0.0001	****
MoCA	19.42 ± 3.42	26.79 ± 0.78	< 0.0001	****
Dementia level	0.53 ± 0.50	0.03 ± 0.16	< 0.0001	****
HAM-A	17.09 ± 7.61	13.68 ± 6.61	0.0538	Non-significant
HAMD-17	10.21 ± 6.47	8.26 ± 5.00	0.2749	Non-significant
PDSS	115.93 ± 19.96	118.61 ± 20.73	0.4236	Non-significant
UPDRS-III	20.92 ± 9.03	16.25 ± 7.67	0.0272	*

^*^P < 0.05. PD: Patients with Parkinson’s disease but without cognitive impairment; PD-CI: patients with Parkinson’s disease and cognitive impairment; H&Y: Hoehn & Yahr; MMSE: Mini-Mental State Examination; MoCA: Montreal Cognitive Assessment; HAM-A: Hamilton Anxiety Scale; HAMD-17: Hamilton Depression Scale-17; PDSS: Parkinson’s Disease Sleep Scale; UPDRS: Unified PD Rating Scale.

### CI increased the severity of PD symptoms

Several questionnaires were implemented to assess various aspects of the quality of life and neuropsychological status of study subjects [Figure 1B and Table 1]. The MMSE and MoCA are important questionnaires for evaluating CI. Based on our research, the PD-CI group showed a considerably lower MMSE score than the PD group (*P* < 0.001). Other than motor symptoms, most patients with PD gradually developed autonomic dysfunction, depression, and sleep disturbances. Therefore, HAM-A and HAMD results showed that the PD-CI group had higher depression and anxiety scores than the PD group, although the differences were not statistically significant. The overall conditions (assessed by UPDRS) were significantly better in the PD group than the PD-CI group (Wilcoxon test, *P* = 0.027, Figure 1B). These findings suggest that the occurrence of CI may make PD patients’ quality of life worse.

### Differences in brain structures in PD and PD-CI groups

**Figure 2:**
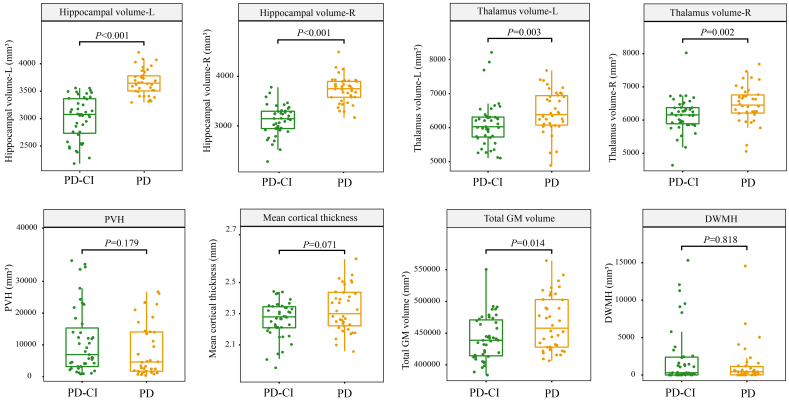
Differences in brain MRI scans between groups. Significant differences were found in Wilcoxon tests, and the corresponding *P* values are shown. PD: PD patients with normal cognition; PD-CI: PD patients with cognitive impairment; R: right; L: left; DWMH: deep white matter hyperintensity; GM: gray matter; PVH: periventricular hyperintensity; MRI: magnetic resonance imaging.

The human brain has extremely complex networks. Many functions related to cognition are related to these networks. Therefore, to explore the difference in brain structure between the two groups, MRI scans were performed. Our results showed that the PD-CI group’s left and right hippocampal, thalamic, and total gray matter volumes are significantly smaller than those of the PD group (Wilcoxon test, *P* < 0.05). Between the two groups, there were no discernible changes in cortical thickness, PVH, or DWMH [Figure 2 and Table 2].

**Table 2:** Differences in MRI results between PD and PD-CI groups

**Index**	**PD-CI**	**PD**	***P***	**Significant difference, Wilcoxon test**
Hippocampal volume-Left (mm^3^)	3,009.63 ± 389.71	3,663.99 ± 233.33	< 0.0001	****
Hippocampal volume-Right (mm^3^)	3,110.81 ± 304.87	3,729.91 ± 269.84	< 0.0001	****
Mean cortical thickness (mm)	2.26 ± 0.12	2.33 ± 0.15	0.0705	Non-significant
Thalamus volume-Left (mm^3^)	6,107.18 ± 665.29	6,459.24 ± 621.93	0.0034	**
Thalamus volume-Right (mm^3^)	6,154.53 ± 517	6,488.69 ± 549.61	0.0021	**
Total gray matter volume (mm^3^)	442,291.69 ± 34,858.14	466,231.84 ± 43,389.97	0.0135	*
PVH (mm^3^)	11,219.12 ± 10,559.17	8,419.13 ± 8,473.85	0.1790	Non-significant
DWMH (mm^3^)	2,261.37 ± 3,844.67	1,307.04 ± 2,683.05	0.8183	Non-significant

^*^P < 0.05. ^**^P < 0.01. PD: Patients with Parkinson’s disease but without cognitive impairment; PD-CI: patients with Parkinson’s disease and cognitive impairment; MRI: magnetic resonance imaging; PVH: periventricular hyperintensity; DWMH: deep white matter hyperintensity.

### Differences in gut microbiota between PD-CI and PD groups

**Figure 3:**
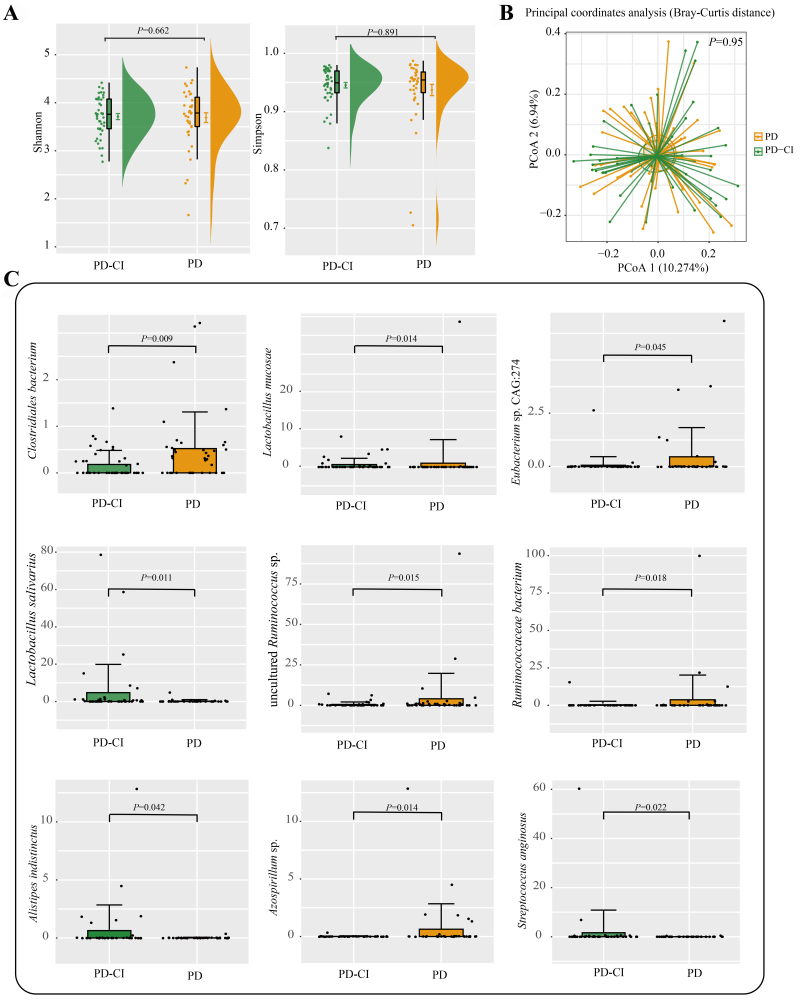
Comparison of gut microbial diversity and composition between groups. (A) Shannon and Simpson diversity indexes and (B) score plot of PCoA (Bray-Curtis distance) of fecal microbiota of PD and PD-CI groups; (C) Differential taxa identified between the two groups of patients. Statistical differences were identified using Wilcoxon tests. Boxplots show median values with upper and lower quartiles. PD: PD patients with normal cognition; PD-CI: PD patients with cognitive impairment; PCoA: principal coordinate analysis.

No notable variations were observed in the Shannon and Simpson diversity indexes between the two groups [Figure 3A]. It suggests that there were no obvious inter-group differences in the microbial diversity and richness of subjects’ fecal microbiota. PCoA revealed that there was no significant intra-group beta-diversity difference between groups [Figure 3B]. Then, the Wilcoxon test was used to compare the differences in SGBs. Nine SGBs showed significant differences across groups. Compared with the PD-CI group, the PD group had significantly higher levels of *Clostridiales bacterium*, *Eubacterium* sp. CAG:274, *Ruminococcaceae bacterium*, and *Azospirillum* sp., whereas an opposite trend was observed for *Lactobacillus salivarius*, *Alistipes indistinctus*, and *Streptococcus anginosus* (Wilcoxon test, *P* < 0.05; Figure 3C, Table 3). Notably, some SCFA-producing bacteria, such as *Ruminococcaceae*, were more abundant in the PD group, whereas *Streptococcus anginosus*, a pathogen, was more abundant in the PD-CI group.

**Table 3:** Significantly different SGBs identified between PD and PD-CI groups

**SGBs**	**Taxonomy**	**Mean_PD-CI**	**Mean_PD**	**SD_PD-CI**	**SD_PD**	***P*-value, Wilcoxon test**
Sample_LPA21.bin_22	*Clostridiales bacterium*	0.18	0.52	0.30	0.79	0.01
Sample_LPA09.bin_1	*Lactobacillus salivarius*	4.75	0.22	15.15	0.79	0.01
Sample_LPA10.bin_4	*Lactobacillus mucosae*	0.66	1.02	1.66	6.27	0.01
Sample_SPA32.bin_3	*Azospirillum* sp.	0.01	0.63	0.05	2.21	0.01
Sample_LPA13.bin_3	uncultured *Ruminococcus* sp.	0.56	4.05	1.52	15.72	0.01
Sample_SPA06.bin_8	*Ruminococcaceae bacterium*	0.36	3.69	2.35	16.51	0.02
Sample_SPA41.bin_3	*Streptococcus anginosus*	1.64	0.02	9.21	0.09	0.02
Sample_SPA08.bin_5	*Alistipes indistinctus*	0.22	0.07	0.64	0.38	0.04
Sample_SPA32.bin_7	*Eubacterium* sp. CAG:274	0.07	0.46	0.40	1.37	0.05

PD: Patients with Parkinson’s disease but without cognitive impairment; PD-CI: patients with Parkinson’s disease and cognitive impairment; SGBs: species-level genomic bins.

### Differences in predicted metabolites between PD-CI and PD groups

**Figure 4:**
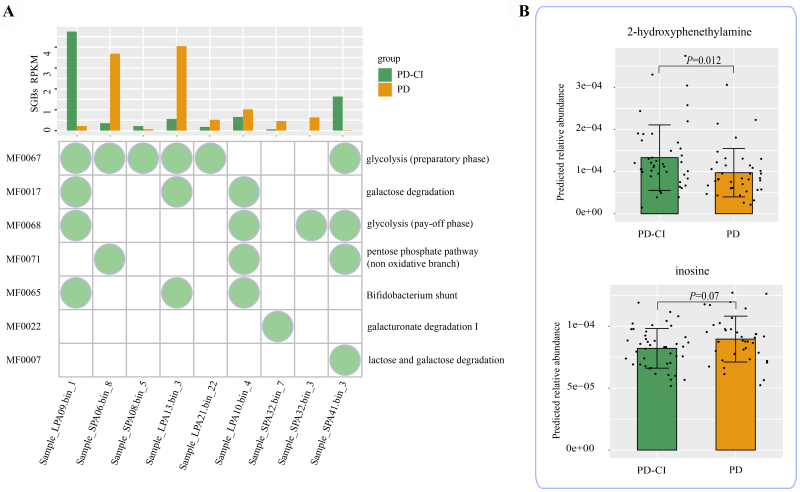
GMMs and predicted metabolites in PD and PD-CI. (A) Distribution of different GMMs in significantly differential SGBs identified between PD and PD-CI groups; (B) Differences in predicted metabolites, compared by Wilcoxon test. The corresponding *P* values are reported. The circles in the bubble plot indicate the inclusion of that module. PD: PD patients with normal cognition; PD-CI: PD patients with cognitive impairment. RPKM: reads per kilobase per million mapped reads; GMMs: gut metabolic modules; SGBs: species-level genomic bins.

MelonnPan analysis found that the PD-CI group was enriched in SGBs related to lactose and galactose degradation, while the PD group had more SGBs involved in galacturonate degradation I [Figure 4A]. Moreover, the PD-CI group was found to have a significantly higher predicted presence of 2-hydroxyphenethylamine compared to the PD group (Wilcoxon test, *P* = 0.012), and there was no significant difference in inosine between the two groups [Figure 4B].

### Gut microbiota in PD and CI

**Figure 5:**
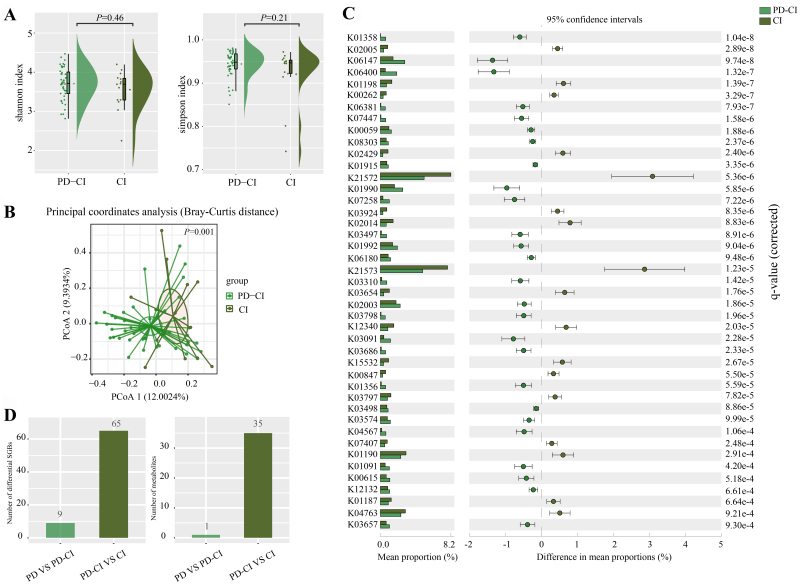
Fecal microbiota in PD patients and in individuals with CI. (A) Shannon and Simpson diversity indexes and (B) score plot of PCoA of PD-CI and CI groups; (C) Significant differentially abundant predicted KEGG metabolic pathways encoded in the fecal microbiota between groups; (D) Number of differentially abundant microbial marker taxa and predicted metabolites between groups. PD: PD patients with normal cognition; PD-CI: PD patients with CI; CI: individuals with CI but not diagnosed with PD; PCoA: principal coordinate analysis; KEGG: Kyoto Encyclopedia of Genes and Genomes.

To further investigate the contribution of gut microbiota in CI, we downloaded fecal metagenomic data from 14 non-PD patients with CI (CI group, MoCA < 26). We compared the baseline information of the PD-CI (*n* = 43) and CI groups. We did not find any significant differences in MoCA and years of education regardless of patient’s CI status [Table 4]. The fecal microbiota of the two groups had no significant difference in alpha diversity [Figure 5A]. However, the PCoA (Bray-Curtis distance) detected a difference in beta diversity (*P =* 0.001, adonis; Figure 5B). When comparing the annotated KEGG pathways of fecal metagenomes, 43 significant differential pathways were identified between the PD-CI and CI groups (Figure 5C, *P* < 0.001). The fecal metagenome of the PD-CI group was significantly enriched in the Pentose phosphate pathway. The CI group was enriched in Sphingolipid metabolism, and nucleotide sugar metabolism [Table 5]. Comparative analysis of differential SGBs in their metagenomes revealed that PD had a greater impact on gut microbiota than CI. More differential SGBs and predicted metabolites were identified when fecal metagenomic datasets of PD-CI and CI groups (65 differential bacteria and 35 predicted metabolites, Figure 5D) were compared with PD and PD-CI groups (9 differential bacteria and 1 predicted metabolite, Figure 5D). Permutational multivariate analysis of variance supported the concept that PD affected gut microbiota more than CI (F = 2.5358, *P* = 0.001 and F = 0.7116, *P* = 0.945, respectively).

**Table 4:** Comparison of basic parameters between CI and PD-CI groups

**Index**	**CI**	**PD-CI**	***P***	**Significant differences, Wilcoxon test**
Age	79.79 ± 6.31	69.07 ± 6.73	< 0.0001	****
MMSE	26.71 ± 2.26	22.19 ± 3.30	< 0.0001	****
MoCA	19.43 ± 2.41	19.42 ± 3.42	0.9554	Non-significant
Education	9.64 ± 3.73	8.65 ± 2.39	0.2918	Non-significant

PD: Patients with Parkinson’s disease but without cognitive impairment; CI: non-Parkinson’s disease patients that had cognitive impairment; MMSE: Mini-Mental State Examination; MoCA: Montreal Cognitive Assessment.

**Table 5:** Annotated KEGG pathways

	**First-level pathways**	**Second-level pathways**	**Third-level pathways**
**K00615**	Metabolism	Carbohydrate metabolism	Pentose phosphate pathway
**K01187**	Metabolism	Carbohydrate metabolism	Carbohydrate metabolism
**K12132**	BRITE hierarchies	Protein families: metabolism	Protein kinases
**K01091**	Metabolism	Carbohydrate metabolism	Glyoxylate and dicarboxylate metabolism
**K07407**	Metabolism	Carbohydrate metabolism	Galactose metabolism
**K01190**	Metabolism	Lipid metabolism	Sphingolipid metabolism
**K03498**	BRITE hierarchies	Protein families: signaling and cellular processes	Transporters
**K03574**	BRITE hierarchies	BRITE hierarchies	DNA repair and recombination proteins
**K04567**	BRITE hierarchies	BRITE hierarchies	Amino acid-related enzymes
**K03797**	BRITE hierarchies	Protein families: metabolism	Peptidases and inhibitors
**K00847**	Metabolism	Carbohydrate metabolism	Amino sugar and nucleotide sugar metabolism
**K01356**	BRITE hierarchies	Protein families: metabolism	Peptidases and inhibitors
**K03310**	Not included in pathway or BRITE	Unclassified: signaling and cellular processes	Transport
**K15532**	Not included in pathway or BRITE	Unclassified: metabolism	Unclassified: metabolism
**K03654**	Genetic information processing	Folding, sorting and degradation	RNA degradation
**K12340**	Environmental information processing	Membrane transport	Bacterial secretion system
**K03686**	BRITE hierarchies	Protein families: genetic information processing	Chaperones and folding catalysts
**K03798**	BRITE hierarchies	Protein families: metabolism	Protein families: metabolism
**K03091**	BRITE hierarchies	Protein families: genetic information processing	Transcription machinery
**K01992**	BRITE hierarchies	BRITE hierarchies	Transporters
**K02003**	BRITE hierarchies	Protein families: signaling and cellular processes	Transporters
**K02014**	BRITE hierarchies	Protein families: signaling and cellular processes	Transporters
**K00059**	Metabolism	Lipid metabolism	Fatty acid biosynthesis
**K21573**	BRITE hierarchies	BRITE hierarchies	Transporters
**K03924**	Not included in pathway or BRITE	Not included in pathway or BRITE	Not included in pathway or BRITE
**K06180**	BRITE hierarchies	Protein families: genetic information processing	Ribosome biogenesis
**K03497**	BRITE hierarchies	Protein families: genetic information processing	Transcription factors
**K07258**	Metabolism	Glycan biosynthesis and metabolism	Peptidoglycan biosynthesis
**K01990**	BRITE hierarchies	Protein families: signaling and cellular processes	Transporters
**K21572**	BRITE hierarchies	Protein families: signaling and cellular processes	Protein families: signaling and cellular processes
**K01915**	Metabolism	Carbohydrate metabolism	Glyoxylate and dicarboxylate metabolism
**K08303**	BRITE hierarchies	BRITE hierarchies	Peptidases and inhibitors
**K02429**	BRITE hierarchies	Protein families: signaling and cellular processes	Transporters
**K07447**	BRITE hierarchies	BRITE hierarchies	Ribosome biogenesis
**K00262**	Metabolism	Amino acid metabolism	Amino acid metabolism
**K06381**	Not included in pathway or BRITE	Unclassified: signaling and cellular processes	Cell growth
**K06400**	Not included in pathway or BRITE	Unclassified: signaling and cellular processes	Cell growth
**K01198**	Metabolism	Carbohydrate metabolism	Amino sugar and nucleotide sugar metabolism
**K02005**	Not included in pathway or BRITE	Not included in pathway or BRITE	Structural proteins
**K06147**	BRITE hierarchies	Protein families: signaling and cellular processes	Transporters
**K01358**	BRITE hierarchies	Protein families: metabolism	Peptidases and inhibitors
**K04763**	BRITE hierarchies	Protein families: genetic information processing	Chromosome and associated proteins
**K03657**	BRITE hierarchies	Protein families: genetic information processing	DNA repair and recombination proteins

KEGG: Kyoto Encyclopedia of Genes and Genomes.

## DISCUSSION

PD mostly affects individuals in the middle to late age. CI, a common complication of PD, affects the daily living activities of patients and brings a burden to their families. The present study analyzed data obtained from neuropsychological questionnaires, brain MRI scans, and fecal metagenomes of PD patients with and without CI, as well as metagenomic data from individuals with CI but not with PD.

Firstly, a few differences were found in the studied parameters between PD-CI and PD groups. In the PD-CI group, there was a substantial decrease in the thickness of the cerebral cortex, the volume of the hippocampus, gray matter, thalamus, and fecal SCFA-producing bacteria. Whether these observations are related to CI remains to be confirmed. Our research discovered that CI may have an impact on PD severity. The gut microbiota has been proposed as a factor that aggravates disease development and pathophysiology. However, our analysis suggested that CI is a less influential factor than PD in affecting patients’ gut microbiota. This study has provided valuable insights into the interplay among gut microbiota, CI, and PD, and identifies future research questions to explore the pathogenesis of PD and CI and their association with gut microbiota.

CI is a frequent complication of PD that significantly impacts quality of life. Therefore, we used the MoCA score to measure cognitive function and explored the neuropsychological functions in our study groups. HADM-17 is commonly used for clinical assessment of anxiety and depression. The HAM-A mainly reflects the anxiety state of patients, including physical and mental anxiety. We observed significantly higher HAM-A and UPDRS scores in the PD-CI group, suggesting that CI could aggravate anxiety symptoms and the severity of PD. The MRI results revealed that the left/right hippocampal volume, left/right thalamus volume, and total gray matter volume were markedly reduced in the PD-CI group. The reduction in the volume of cognitive-related brain regions is considered a marker of neurodegeneration^[28]^. The atrophy of some key areas of human brain can cause a decline in cognitive ability. Therefore, our results indicate that the altered brain structure in PD-CI, as compared to PD, could explain the loss of cognitive function.

A previous study explored the link between gut microbiota and PD and proposed that the development of PD could be at least partly caused by intestinal inflammation^[29]^. Clinical research has also reported that *Blautia producta* was deficient in PD patients and the level of fecal butyrate was lower, which negatively correlated with disease severity^[30]^. Therefore, the gut microbiota is thought to relate to the development of PD because it regulates colonic and body inflammation. In the present study, the comparison of the fecal microbiota composition and structure between the PD-CI and PD groups did not reveal any significant variations. However, the beta diversity of the PD-CI and CI groups showed significant differences.

A prior study conducted in China found that PD patients had a higher gut bacterial richness and diversity compared to healthy controls. It was also accompanied by an altered gut microbiota structure and enrichment in Genera *Clostridium IV*, *Aquabacterium*, *Holdemania*, *Sphingomonas*, *Clostridium XVIII*, *Butyricicoccus*, and *Anaerotruncus*^[31]^. Even in two PD cases, the gut microbiota is observed to alter at the time of diagnosis - SCFAs-producing bacteria were less abundant^[2]^. Another study conducted on middle-aged and elderly people reported that mild CI would alter the gut microbial abundance for species such as *Prevotella ruminicola*, *Bacteroides thetaiotaomicron*, and *Bacteroides xylanisolvens.* The authors argued that these microbes may contribute to the development of mild CI^[32]^. Therefore, there is a consensus that fecal microbiota differs significantly between PD and CI and healthy individuals. Notably, Bonham reported that differences in microbiota taxa and genes were associated with overall cognitive function and the size of brain regions. In healthy children, they examined the association of microbiota with neuroanatomy and cognition^[33]^. In sleep deprivation mouse model, it is found that intestinal flora disorder can lead to CI. The intestinal flora stimulating excessive phagocytosis of hippocampal protrusion proteins by microglia is suggested to be the potential mechanism for that effect^[34]^. Studies have suggested that the effect of *Clostridium butyricum* on cognitive function is mainly due to its ability to reshape gut microbiota. Additionally, it is suggested to be a protective effect on obesity-related CI and neurodegeneration through the microbiota-gut-brain axis^[35]^. Therefore, it would be of further interest to investigate if alterations in the gut microbiota or abundance of specific microbes have a direct role in PD pathogenesis and cognitive performance. Based on our results, PD has a greater impact on the gut microbiota compared to CI. However, the causal relationship between gut microbiota and CI in PD remains to be understood. In the future, multi-omics technology should be used to further explore such a relationship and potential mechanism.

It has also been discovered that gut metabolites impact cognitive performance. For example, a previous study has reported that the gut-brain axis may influence the blood-brain barrier and trigger neuroinflammation, which might lead to dementia and CI^[36]^. The study found that the transplantation of the gut microbiota of Alzheimer’s disease (AD) mouse model into normal mice could lead to intestinal inflammation. Consequently, it increased brain inflammation, inhibited neural activity in the hippocampus, and resulted in the impairment of learning and memory abilities^[37]^. Another class of gut metabolites that is crucial in reducing neuroinflammation is SCFAs. An animal study found that the species *Roseburia hominis* could reduce neuroinflammation by producing acetic acid and butyric acid to inhibit histone deacetylases^[38]^_._ Butyrate is a SCFA that can affect the central nervous system by changing the expression of brain-derived neurotrophic factors. Although the levels of SCFAs in our study subjects were not measured, we found that the abundance of sequences representing fecal SCFA-producing bacteria decreased and sequences representing pathogens increased in PD-CI cases. These observations are consistent with a previous cohort study which reported a decrease in fecal but not plasma levels of SCFAs in PD patients. Furthermore, the fecal and plasma SCFA levels were associated with motor and cognitive function and specific changes in gut microbiota^[39]^. It is argued that low SCFA is associated with poor cognitive performance. Compared with the healthy people, in the PD group, low SCFAs in PD were significantly associated with poorer cognition^[40]^. Another study involving 165 PD patients used the random forest model to analyze gut microbiota and accurately predicted the disease progression within two years. A decrease in the abundance of SCFA-producing bacteria and an increase in mucin-degrading bacteria were indicative of a faster disease progression^[41]^. Thus, SCFAs may play an important role in the development of CI, and our findings consistently suggest a correlation between PD and CI and the abundance of SCFA-producing bacteria. However, the specific relationship needs to be further explored. In the future, large-scale clinical trials are required to conduct quantitative and qualitative analyses of SCFAs in intestines and blood to comprehensively explore the role of SCFAs in CI.

Our study also identified 43 differential abundance pathways between PD-CI and CI groups. It is worth mentioning that the PD-CI group was significantly enriched in the Pentose phosphate pathway, while the CI group was enriched in Sphingolipid metabolism and nucleotide sugar metabolism. The study found that the polysaccharide biosynthesis and metabolism-related pathways were increased in AD and MCI patients, and the immune system-related pathways were decreased in AD patients^[42]^. Therefore, the occurrence of CI is related to the changes in related metabolic pathways. The above results provide new insights into the potential of gut microbiota modification in the treatment of CI.

### Limitation

First of all, this is a data reanalysis of a relatively small sample size, and therefore, the drawn conclusion may not be universally applicable. Second, this study only analyzed the intestinal flora, metabolic pathways, and predicted metabolites. Therefore, data should be combined with the results of flora metabolites, blood metabolites, and animal studies to explore underlying pathophysiological mechanisms. Finally, our data explored the relationship between CI and patient demographics but did not account for the confounding factors, such as diet and economic factors. These factors may affect gut microbiota and should be considered in future studies.

### Conclusion

The present research discovered variations in the structure and composition of gut microbiota in individuals with PD, CI, and PD-CI. The abundance of gene sequences representing SCFA-producing bacteria decreased in the fecal metagenomes of PD-CI patients, while those representing pathogenic bacteria increased. The metabolic pathways encoded in the fecal metagenome also altered in individuals with CI as compared to PD-CI. We also found that CI may aggravate the severity of PD, although it did not induce significant alterations in the gut microbiota of the study participants.

## DECLARATIONS

### Availability of data and materials

All metagenomic data can be retrieved from the NCBI database.

### Copyright

© The Author(s) 2024.
